# Integrative Genomic Analysis Identifies Isoleucine and CodY as Regulators of *Listeria monocytogenes* Virulence

**DOI:** 10.1371/journal.pgen.1002887

**Published:** 2012-09-06

**Authors:** Lior Lobel, Nadejda Sigal, Ilya Borovok, Eytan Ruppin, Anat A. Herskovits

**Affiliations:** 1 The Department of Molecular Microbiology and Biotechnology, The George S. Wise Life Sciences Faculty, Tel Aviv University, Tel Aviv, Israel; 2 The Sackler Faculty of Medicine, Tel Aviv University, Tel Aviv, Israel; 3 The Blavatnik School of Computer Sciences, Tel Aviv University, Tel Aviv, Israel; University of Geneva Medical School, Switzerland

## Abstract

Intracellular bacterial pathogens are metabolically adapted to grow within mammalian cells. While these adaptations are fundamental to the ability to cause disease, we know little about the relationship between the pathogen's metabolism and virulence. Here we used an integrative Metabolic Analysis Tool that combines transcriptome data with genome-scale metabolic models to define the metabolic requirements of *Listeria monocytogenes* during infection. Twelve metabolic pathways were identified as differentially active during *L. monocytogenes* growth in macrophage cells. Intracellular replication requires *de novo* synthesis of histidine, arginine, purine, and branch chain amino acids (BCAAs), as well as catabolism of L-rhamnose and glycerol. The importance of each metabolic pathway during infection was confirmed by generation of gene knockout mutants in the respective pathways. Next, we investigated the association of these metabolic requirements in the regulation of *L. monocytogenes* virulence. Here we show that limiting BCAA concentrations, primarily isoleucine, results in robust induction of the master virulence activator gene, *prfA*, and the PrfA-regulated genes. This response was specific and required the nutrient responsive regulator CodY, which is known to bind isoleucine. Further analysis demonstrated that CodY is involved in *prfA* regulation, playing a role in *prfA* activation under limiting conditions of BCAAs. This study evidences an additional regulatory mechanism underlying *L. monocytogenes* virulence, placing CodY at the crossroads of metabolism and virulence.

## Author Summary

Intracellular bacterial pathogens have developed sophisticated mechanisms to invade and replicate within eukaryotic cells. For successful replication, pathogens have adapted metabolically to the intracellular niche. While this adaptation is fundamental to the ability to cause disease, we know little about pathogen's intracellular metabolism and its association with virulence. In this study we took a global approach that combines computational and experimental methods to decipher the intracellular metabolic requirements of the human bacterial pathogen *Listeria monocytogenes*. We identified 12 metabolic pathways to be differentially active during infection in comparison to growth in rich lab media. We validated the essentiality of the active pathways for *L. monocytogenes* intracellular replication. Pathways included: biosynthesis of histidine, arginine, purine, and branch chain amino acids (BCAAs), as well as the catabolism of L-rhamnose and glycerol. Next we analyzed whether the requirement for these nutrients associates with virulence. We found that limiting concentrations of BCAAs, primarily of isoleucine, results in robust induction of the bacterial virulence state, a response that is dependent on the isoleucine responsive regulator, CodY. CodY was responsible for the up-regulation of the major virulence regulator of *L. monocytogenes*, PrfA. This study supports the premise that pathogens metabolism and virulence are closely interlinked.

## Introduction

Intracellular bacterial pathogens have developed sophisticated mechanisms to enter eukaryotic cells and replicate within them. These mechanisms involve bacterial proteins that overcome host defense strategies and barriers as well as nutritional limitations. Intracellular pathogens are generally categorized into two groups based on the compartment where they replicate, the vacuole/phagosome or the host cell cytosol. Each intracellular niche presents unique nutritional challenges demanding that bacteria exhibit specific metabolic adaptations to proliferate successfully. Vacuolar pathogens, such as *Legionella pneumophilla* and *Mycobacterium tuberculosis*, actively modify their compartment via secretion of effector proteins that enrich the vacuole with nutrients to support growth; nevertheless growth rate in the vacuole is much slower than in rich media. In contrast, intracellular cytosolic bacteria, such as *Listeria monocytogenes*, *Sheigella felxenri* and *Burkholderia pseudomallei* manage to exploit their niche such that growth rates resemble growth in rich media [1]. Little is known about the metabolic adaptations that enable intracellular cytosolic pathogens to grow rapidly or if such adaptations affect virulence. A better understanding of how these bacteria overcome nutritional limitations will give insight into cytosol nutrient composition and could facilitate development of drugs against intracellular pathogens.

*L. monocytogenes* is a Gram-positive facultative intracellular bacterial pathogen and the causative agent of listeriosis in humans, a disease with a variety of clinical manifestations including meningitis and abortion [2]. *L. monocytogenes* infects phagocytic and non-phagocytic cells, using surface expressed proteins called internalins, which bind and induce bacterial uptake by endocytosis [3]. Upon entry, *L. monocytogenes* escapes from the phagosome/vacuole into the host cytosol by producing the pore-forming hemolysin toxin, listeriolysin O (LLO, encoded by the *hly* gene), and two additional phospholipases [4]–[6]. Once in the host cytosol, *L. monocytogenes* multiplies rapidly and expresses the surface protein, ActA, which recruits the host actin polymerization machinery to propel the bacteria in the cytosol and facilitate spread from cell to cell [7], [8]. All known virulence factors involved in internalization, vacuolar escape and cell-to-cell spread are co-regulated by the major virulence activator, PrfA [9].

*L. monocytogenes* uses several carbon sources during intracellular growth, but primarily glycerol, di-hydroxyacetone and phosphorylated carbohydrates (such as glucose 1-phosphate), indicating the availability of these substrates in the cytosolic niche [10]–[12]. Glycerol uptake is mediated by a glycerol permease, whereas phosphorylated sugars are transported via the specialized hexose-phosphate transporter, Hpt. Both systems are induced intracellularly and are important for bacterial replication [11], [13]. It is well established that carbon metabolism during intracellular growth is linked directly to the virulence of *L. monocytogenes*. Carbohydrates transported via the phosphoenolpyruvate phosphotransferase system (PTS) (such as glucose and cellobiose) repress the activity of PrfA whereas non-PTS carbon sources (such as glycerol and glucose 1-phosphate that are available in the host cytosol) induce PrfA activity, resulting in elevated expression of virulence genes [reviewed in: [14] and [15]]. Based on these observations, it was proposed that non-PTS sugars might serve to signal *L. monocytogenes* of its intracellular location.

Various additional metabolic pathways were indicated as important for intracellular replication of *L. monocytogenes*, including several amino acid biosynthesis pathways such as the branch chain amino acid (BCAA) and arginine pathways [16], [17], the common aromatic compounds biosynthesis (shikimate) pathway, as well as the synthesis and uptake of thiamine (vitamin B_1_) [18], [19]. A special adaptation of *L. monocytogenes* to the cytosolic niche is its ability to obtain the co-factor lipoate from the host, as it cannot be synthesized by the bacteria. Listerial expression of a lipoate ligase, LplA1, enables the co-factor to be derived from host lipoyl-peptides [20]. As for nitrogen sources, it is thought that *L. monocytogenes* utilizes ammonium, arginine and ethanolamine [21], [22]. The latter is highly abundant in mammalian cells as it is the breakdown product of phosphatidylethanolamine. The ability to use ethanolamine as a nitrogen and/or carbon source is linked to the pathogenesis of several bacteria, such as *Salmonella*, *Enterococcus* and *Clostridium* [22]. Like *Salmonella*, listerial genomes encode the ethanolamine utilization pathway [23]. Taken together, these various adaptations highlight the complex metabolic requirements of intracellular growth.

Recent advances in genomic sequencing and development of constraint-based metabolic models enable now the reconstruction of genome scale metabolic networks of different organisms. This approach enables a better comprehension of the complete metabolic network of an organism under different growth conditions as well as the prediction of essential metabolic genes [24], [25]. These remarkable advances have prompted several studies of the metabolic networks of pathogens during infection, with the goal of characterizing potential drug targets [26]–[30]. Lately, as part of the SEED project an automated genome-scale metabolic model of *L. monocytogenes* metabolism has been developed [31] that comprises a stoichiometric matrix of reactions and metabolites representing the organism's entire metabolic network. In this study we used this metabolic model to analyze the global metabolic state of *L. monocytogenes* during infection.

**Figure 1:**
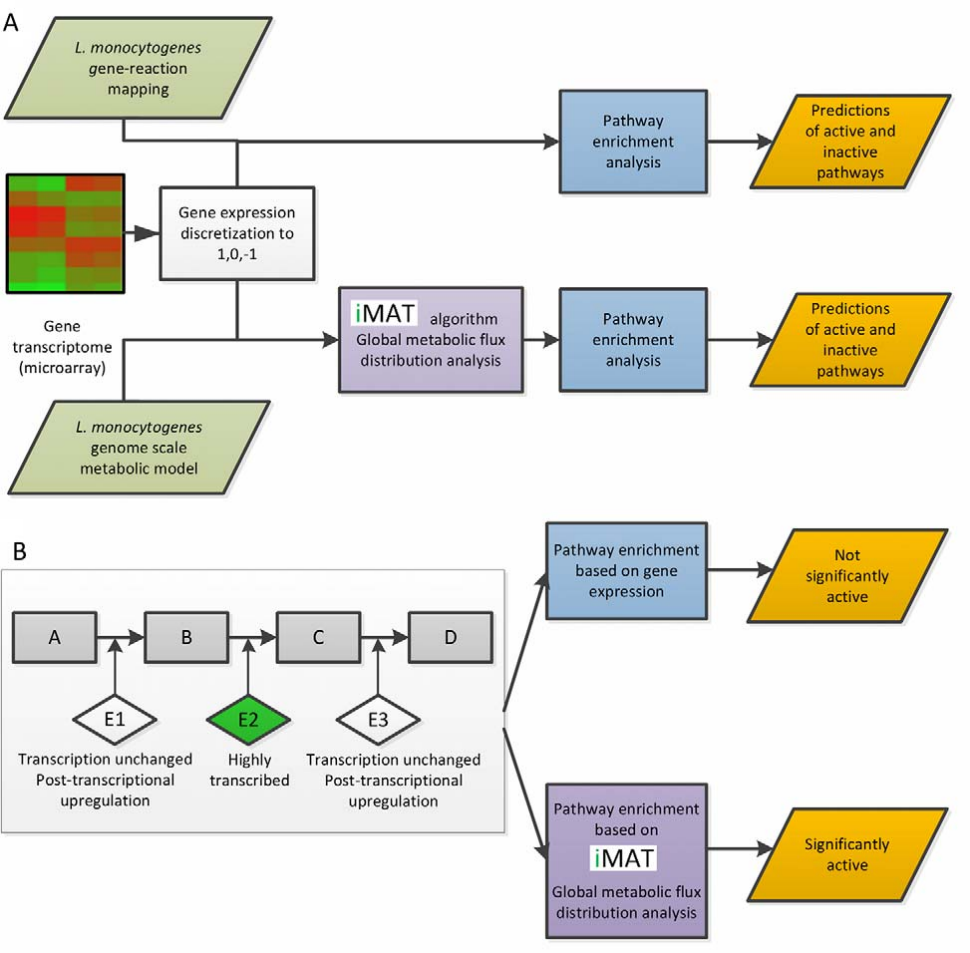
Workflow illustration. A. A presentation of the study workflow, indicating the two methods of analysis that were used. B. An example of iMAT's ability to predict post-transcriptional regulation. This example represents a case of which only one of the three enzymes of a metabolic pathway is transcriptionally induced, while the other two are regulated post-transcriptionally. iMAT algorithm takes into a count the global metabolic fluxes according to the transcriptomic data and can correctly predict whether an entire pathway is up regulated, if it is in line with the flux distribution in the model. Rectangles represent metabolites, diamonds represent enzymes.

To model the metabolic state of an organism under a given set of conditions, the availability of nutrients needs to be simulated. This information is mostly unknown for complex systems, but a specific metabolic state can be specified by integrating relevant transcriptome, proteome or metabolome (‘omics’) data into the metabolic model. A computational tool that performs this data integration was recently developed, the integrative Metabolic Analysis Tool (iMAT) [32]. Briefly, taking into account omics data, iMAT predicts flux activity that is stoichiometrically consistent across the metabolic network in a global manner. It determines a subset of reactions to be confidently active or inactive, while leaving pathways that have alternative flux distributions as unknown due to the existence of isozymes or alternative metabolic pathways. By integrating the omics data with a metabolic model encompassing the pertaining biochemical knowledge, iMAT provides a more comprehensive and accurate prediction of flux activity, reflecting the effects of possible post-transcriptional and post-translational regulations that go beyond the information embedded in the raw gene expression data [33]. For example, consider a metabolic pathway composed of three enzymes that are highly active in a given condition. The transcription of only one of the enzymes is up regulated, while the transcription of the other two enzymes remains unchanged. Conventional bioinformatic pathway enrichment analysis based solely on transcriptional data will fail to denote this pathway as highly active, however iMAT analysis could be more successful, if the pathway's activation results in a global network flux distribution that is best consistent with the overall gene expression input (Figure 1).

In this study we applied iMAT analysis to transcriptome data and a genome scale metabolic model of *L. monocytogenes* to better understand the bacterial metabolic requirements during growth in macrophage cells. Using this approach several metabolic pathways were predicted active or inactive during intracellular growth, potentially reflecting the metabolites availability within these cells. We experimentally deleted key genes in the pathways predicted to be active, validating their contribution to *L. monocytogenes* intracellular replication and studied the association of the corresponding metabolites in regulation of *L. monocytogenes* virulence genes.

## Results

### Conventional transcriptome-based pathway enrichment analysis of metabolic pathway changes during intracellular growth

The global transcriptional profiles of *L. monocytogenes* (strain 10403S) growing in two different conditions were assessed using whole genome microarray analysis. RNA was extracted from *L. monocytogenes* grown either inside macrophages (at 6 hours post infection, representing cytosolic replicating bacteria) or in brain heart infusion (BHI) broth (mid log phase). Transcription levels in intracellular bacteria were designated as relative to those in bacteria growing in BHI. Gene expression was coded as 1, −1 or 0 for each gene, indicating a minimum of twofold increase, decrease, or no change, respectively, in transcript levels during intracellular growth versus growth in BHI (Table S1). Next, we retrieved the list of metabolic reactions of *L. monocytogenes* from the automated genome scale metabolic model reconstruction pipeline [31]. This model consists of 1254 reactions and 816 metabolic genes, encompassing 102 SEED metabolic pathway annotations [34]. Genes in the transcriptome array were assigned to metabolic reactions and their expression levels were converted into reaction activity based on the model's built-in gene-reactions mapping matrix (*i.e.* enzymatic complexes and isozymes). We then applied a standard hypergeometric test for pathway enrichment taking into account each metabolic reaction observed to change (up or down) to determine which pathways were enriched for altered activities. A metabolic pathway significantly enriched with up-regulated reactions was considered highly active, while a metabolic pathway enriched with down-regulated reactions was considered inactive (Table 1). In agreement with previous studies of metabolic pathways that contribute to *L. monocytogenes* growth during *in vitro* infection, we identified arginine biosynthesis, branched chain amino acids (BCAA) biosynthesis and glycerol utilization as pathways highly active during intracellular replication [10], [11], [16]. In addition to the known pathways, our analysis predicted two additional pathways as induced intracellularly: the histidine biosynthesis and L-rhamnose utilization pathways. Along with these highly active pathways, we identified two metabolic pathways to be specifically down regulated during intracellular growth: the formaldehyde assimilation genes and the bacterial fatty acids biosynthesis pathway (FASII).

**Table 1:** Metabolic pathways predicted to be differentially active during *L. monocytogenes* intracellular growth by standard bioinformatics analysis and iMAT analysis.

Metabolic pathway	Standard bioinformatics analysis (P-value)^a^	iMAT analysis (P-value)^a^	Prediction	References related to *L. m.* virulence
Arginine biosynthesis	1.25E-05	2.14E-07	Highly active	[16]
Branch-chain amino acids biosynthesis	3.52E-10	7.68E-12	Highly active	[16], [17]
Histidine biosynthesis	1.25E-05	3.68E-09	Highly active	
L-Rhamnose utilization	0.001	2.27E-05	Highly active	[57]
*De novo* purine biosynthesis	NP	5.02E-04	Highly active	[17]
Glycerol utilization	8.77E-06	6.74E-04	Highly active	[10], [11]
Common aromatic compounds biosynthesis	NP	9.51E-03	Highly active	[17], [18]
Fatty acids biosynthesis FASII	0.014	1.86E-17	Inactive	
Pantothenate and CoA biosynthesis	NP	1.73E-06	Inactive	
Mevalonate pathway for isopernoids biosynthesis	NP	2.09E-09	Inactive	[35]
Alternative pathways for isopernoids biosynthesis	NP	1.35E-03	Inactive	[35]
Heme and siroheme biosynthesis	NP	1.85E-03	Inactive	
Formaldehyde assimilation: Ribulose monophosphate pathway	0.002	NP	Inactive	

a - Based on hypergeometric enrichment test.

NP-Not predicted.

### Model-based iMAT analysis of metabolic pathway changes during intracellular growth

Next, we applied the iMAT algorithm to predict metabolic flux activity during intracellular growth of *L. monocytogenes*. We defined a simulated medium that best represents the cytosolic environment (Table S2), successfully yielding a predicted *in silico* generation time of 55 min/gen, similar to the one determined experimentally (57 min/gen). iMAT returns a three-valued output, rating the predicted activity of each reaction as confidently active, confidently inactive, or unknown. This output was analyzed by computing the hypergeometric enrichment score of each pathway based on the predicted tri-valued flux activity of each of its reactions (this time determined by iMAT in a global, model based manner) (Table 1). In line with the literature and our conventional gene expression analysis, the arginine, histidine and BCAA biosynthesis pathways together with L-rhamnose and glycerol utilization pathways were predicted to be highly active during intracellular growth. In addition, however, iMAT analysis identified two other metabolic pathways as active intracellularly: the common aromatic compounds biosynthesis (shikimate) pathway and the *de novo* purine biosynthesis pathway. Notably, the importance of the *de novo* purine biosynthesis pathway was highlighted by iMAT predictions of metabolites that are imported or exported during intracellular growth (based on the global fluxes and directionality of reactions) (Table S3). Indeed, iMAT predicted uptake of all nucleotides from the host, except for adenosine, explaining the need for *de novo* purine biosynthesis. Furthermore, iMAT designated five metabolic pathways as inactive/down regulated: the FASII biosynthesis pathway, in line with our conventional gene expression analysis; the mevalonate and the alternative isopernoids biosynthesis pathways [in agreement with [35]]; and two newly identified pathways, the heme biosynthesis and pantothenate-coenzyme A biosynthesis pathways. Statistical analysis of iMAT accuracy versus conventional gene expression analysis, based on a literature database of metabolic pathways that were reported for their role in *L. monocytogenes* intracellular growth (Table S4), revealed that iMAT predictions are more accurate (P-Value = 0.0001 *vs.* P-Value = 0.052, hypergeometric distribution test). The precision and recall for iMAT analysis are P = 0.583, R = 0.5, compared to that of the gene expression analysis P = 0.428, R = 0.357. Overall iMAT analysis resulted in a predicted metabolic shift in 12 metabolic pathways upon growth of *L. monocytogenes* within macrophages, five of which were missed by conventional gene expression analysis. Notably, both methods predicted the novel contribution of histidine biosynthesis and rhamnose utilization to *L. monocytogenes* intracellular replication.

### Experimental validation of the predicted metabolic pathways induction during intracellular growth

**Figure 2:**
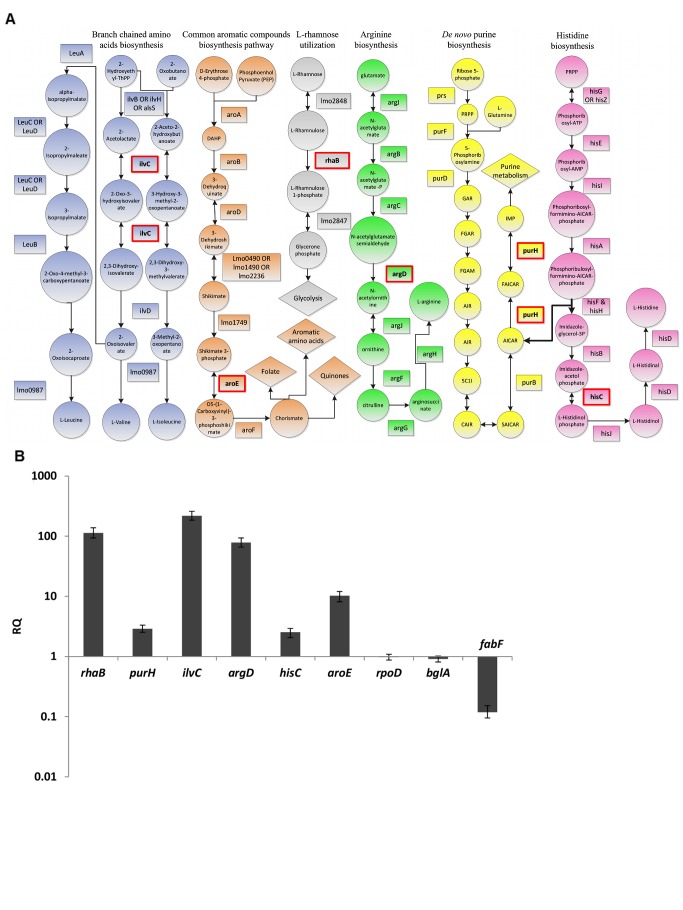
*L. monocytogenes* metabolic pathways predicted to be highly active during intracellular growth. A. Schematic metabolic maps of *L. monocytogenes* pathways predicted to be active during intracellular growth. Circles indicate metabolites, rectangles represent enzymes and diamonds designate downstream pathways. Enzymes that were deleted in this work are denoted by red rectangles. B. RT-qPCR analysis of the indicated transcripts in bacteria growing in macrophages at 6 hours post infection (h.p.i.) *vs.* growing in BHI medium mid-logarithimically. Transcription levels are presented as relative quantity (RQ), relative to levels in BHI. mRNA levels were normalized to 16S rRNA. The data represents 2 biological repeats (N = 2). Error bars indicate a 95% confidence interval.

**Figure 3:**
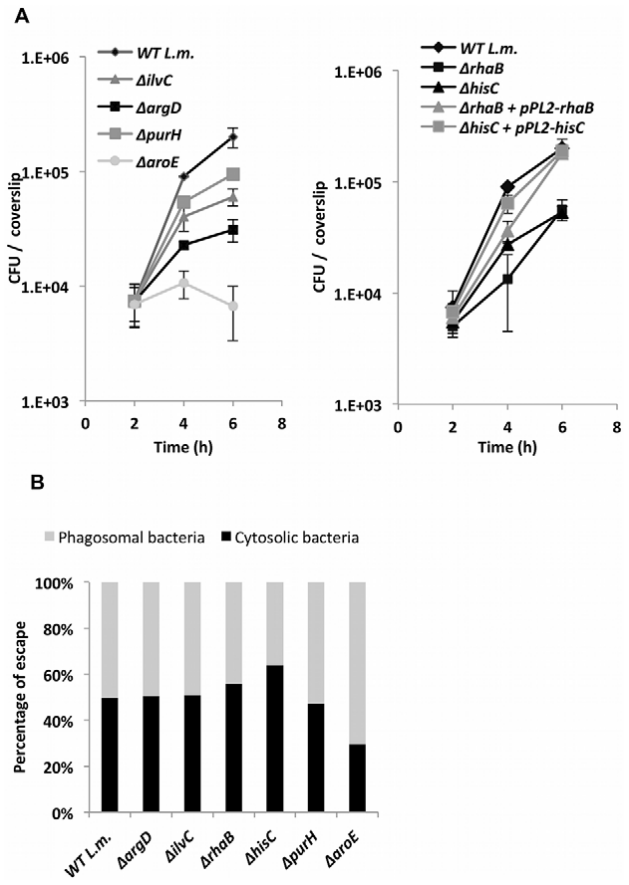
*L. monocytogenes* metabolic mutants grow less efficiently in macrophage cells. A. Intracellular growth curves of WT *L. monocytogenes* and metabolic mutants. Left panel: Δ*ilvC*, Δ*argD*, Δ*purH*, Δ*aroE* mutants (for complemented strains, see Figure S3). Right panel: the newly identified metabolic mutants Δ*rhaB* and Δ*hisC*, and complemented strains harboring a copy of *rhaB* and *hisC* genes on the pPL2 plasmid. The experiment was performed 3 times and representative growth curves are shown. Error bars indicate standard error. B. Percentage of bacteria that escaped macrophage phagosomes at 2.5 h.p.i. as determined by microscope fluorescence assays. The data represents 3 independent experiments (N = 3).

Next, we decided to validate the essentiality of all the metabolic pathways predicted by iMAT analysis to be active during intracellular growth, except for the glycerol utilization pathway that has already been studied in great detail [10], [11]. We chose key listerial enzymes in the active pathways and assessed directly their transcription levels during intracellular growth, as a validation for the microarray data, as well as the consequences of non-polar in- frame deletions of the respective genes. In total, six genes encoding key enzymes from six different metabolic pathways were subjected to further study (Table 2). As depicted by the metabolic map of *L. monocytogenes* all the chosen genes are central and cannot be compensated (Figure 2A). The genes transcription during intracellular growth of *L. monocytogenes* in macrophage cells versus growth in BHI medium was assessed using quantitative real-time PCR (RT-qPCR). All six metabolic genes were up-regulated during intracellular growth whereas *rpoD* and *bglA*, two control genes, remained unchanged. As another control the transcription level of *fabF* gene (*lmo2201*), part of the FASII pathway that was predicted to be down-regulated intracellularly, was analyzed and indeed this gene was shown to be down-regulated during intracellular growth in macrophage cells in comparison to growth in BHI media (Figure 2B). *rhaB*, *ilvC* and *argD* were highly induced, at least 64 fold, while *purH*, *hisC* and *aroE* were induced 2–8 fold (Figure 2B). These enhanced transcriptional levels confirmed that indeed the six pathways are induced and active during *L. monocytogenes* intracellular growth. Next, we knocked out each one of the selected genes in *L. monocytogenes* 10403S strain and analyzed the growth capabilities of the resulting six mutants (Table 2). We first tested whether the mutants are indeed defective in their corresponding metabolic pathways. Mutants were grown in minimal defined medium (MDM) [36] and in MDM media that was specifically depleted of the cognate pathway metabolite (Figure S1). As shown in Figure S1, while most mutants grew normally in MDM media (except for *ΔaroE* and *ΔpurH* mutants) each one demonstrated a growth defect when the metabolite of their targeted pathway was depleted. As expected, introducing back the corresponding genes complemented the growth defects of these mutants. When tested for growth in BHI, all mutants, with the exception of *ΔaroE* (as reported also in [18]), grew like WT bacteria, demonstrating that these metabolic pathways are not essential for growth in rich medium (Figure S2). Next, we assessed the contribution of the predicted pathways to intracellular growth of *L. monocytogenes*. Macrophage cells were infected with the different mutants and intracellular growth was measured. As shown in Figure 3A, all the metabolic mutants replicated less efficiently in macrophage cells in comparison to WT bacteria or to the complemented strains (Figure 3A and Figure S3). The intracellular growth of *ΔaroE* was highly impaired, however as shown before this gene is also required during growth in rich medium (Figure S2 and [18]). To exclude the possibility that the metabolic mutants are defected in phagosomal escape, we performed a microscope based escape assay, and demonstrated that all mutants were able to escape the macrophages phagosomes like WT bacteria, with the exception of *ΔaroE* that had a minor defect (Figure 3B). Taken together, these results validate that each of the six predicted pathways is required for intracellular replication of *L. monocytogenes*, and importantly, establish for the first time a role for histidine biosynthesis and rhamnose catabolism during *L. monocytogenes* intracellular growth.

**Table 2:** The genes selected for knock out encode key enzymes in the metabolic pathways predicted to be active during intracellular growth.

Gene name	LMRG identifier (lmo identifier)	Enzyme description	Pathway
*aroE*	01070.6 (lmo1923)	3-phosphoshikimate 1-carboxyvinyltransferase	Common pathway of aromatic compounds biosynthesis
*purH*	02506.9 (lmo1765)	IMP cyclohydrolase	*De novo* purine biosynthesis
*rhaB*	02420.6 (lmo2849)	rhamnulokinase	L-rhamnose utilization
*hisC*	01072.6 (lmo1925)	histidinol-phosphate aminotransferase	Histidine biosynthesis
*argD*	01379.6 (lmo1588)	acetylornithine aminotransferase	Arginine biosynthesis
*ilvC*	01134.6 (lmo1986)	ketol-acid reductoisomerase	Branched chain amino acids biosynthesis

### Metabolic requirements influence the expression of major virulence genes

**Figure 4:**
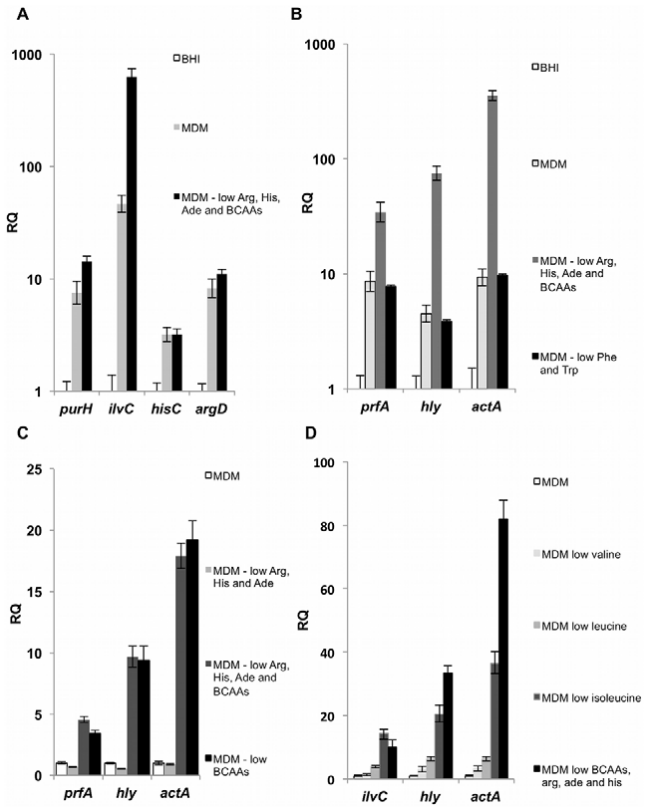
Nutrient availability influences transcription of *L. monocytogenes* metabolic and virulence genes. A. Transcription levels of key metabolic genes during growth of WT *L. monocytogenes* in BHI medium, minimal defined medium (MDM) and MDM with low concentrations (10-fold less) of BCAAs, histidine (His), arginine (Arg) and adenine (Ade). Transcriptional levels are presented as relative quantity (RQ), relative to BHI medium. B. Transcription levels of *prfA*, *hly*, and *actA* virulence genes during growth of WT *L. monocytogenes* in BHI medium, MDM, MDM with low concentrations of BCAAs, His, Arg and Ade and in MDM with low concentrations of phenylalanine (Phe) and tryptophan (Trp) (10 µg ml^−1^). Transcriptional levels are presented as RQ, relative to BHI medium. C. Transcription levels of *prfA*, *hly*, and *actA* virulence genes during growth of WT *L. monocytogenes* in MDM and MDM with low concentrations of the designated nutrients. Transcriptional levels are presented as RQ, relative to MDM. D. Transcription levels of *ilvC*, *hly*, and *actA* genes during growth of WT *L. monocytogenes* in MDM and MDM with low concentrations of the designated nutrients. Transcriptional levels are presented as RQ, relative to MDM. Overnight precultures were grown in MDM media and diluted for growth under the indicated conditions. Bacteria were harvested at O.D._600 nm_ of 0.3, representing exponential growth. mRNA levels were normalized to *rpoD* mRNA. The results represent 3 independent experiments (N = 3). Error bars indicate a 95% confidence interval.

Our analyses established that four anabolic pathways are specifically important during *L. monocytogenes* growth in macrophage cells, namely the BCAA, histidine, arginine and purine biosynthesis pathways. This finding surmises that limited amounts of these nutrients are available in the host cytosol. Given that the abundant availability of non-PTS sugars in the host cytosol is suspected to serve as a signal of intracellular location (see introduction), we investigated if low availability of BCAAs, histidine, arginine and purine might serve similarly as an intracellular signal that modulate the transcription of the virulence genes. Therefore, we tested whether the regulation of these four pathways is linked to the regulation of virulence genes. To this end, WT *L. monocytogenes* was grown in BHI, minimal defined medium (MDM) and MDM with reduced concentrations (10-fold less) of arginine, histidine, adenine and BCAAs (*i.e.* isoleucine, leucine and valine), which aimed to mimic the intracellular free amino acids concentration [37]–[39]. The transcription levels of corresponding metabolic genes and virulence genes (*prfA* the master virulence activator gene, *hly* gene encoding for LLO, and *actA* gene) were then analyzed at mid-log phase. The expression of the metabolic genes (*purH*, *ilvC*, *hisC* and *argD*) was induced during growth in MDM relative to growth in BHI rich medium (Figure 4A), indicating that MDM already contains limiting concentrations of their cognate nutrients. Growth in MDM with ∼10-fold reduced concentrations of arginine, histidine, adenine, isoleucine, leucine and valine resulted in a prolonged lag phase (Figure S4) and in further induction of the BCAA biosynthesis pathway, as shown by increased expression of the *ilvC* gene (Figure 4A). Remarkably, while growth in MDM resulted in moderate up-regulation of the virulence genes compared to BHI, limiting further the concentrations of arginine, histidine, adenine and BCAAs triggered robust induction of all three virulence genes (Figure 4B). This dramatic response was specific to nutrients synthesized by the intracellularly active metabolic pathways, as growth in MDM with reduced concentrations of other amino acids such as phenylalanine and tryptophan had no effect on expression of the virulence genes (Figure 4B).

**Figure 5:**
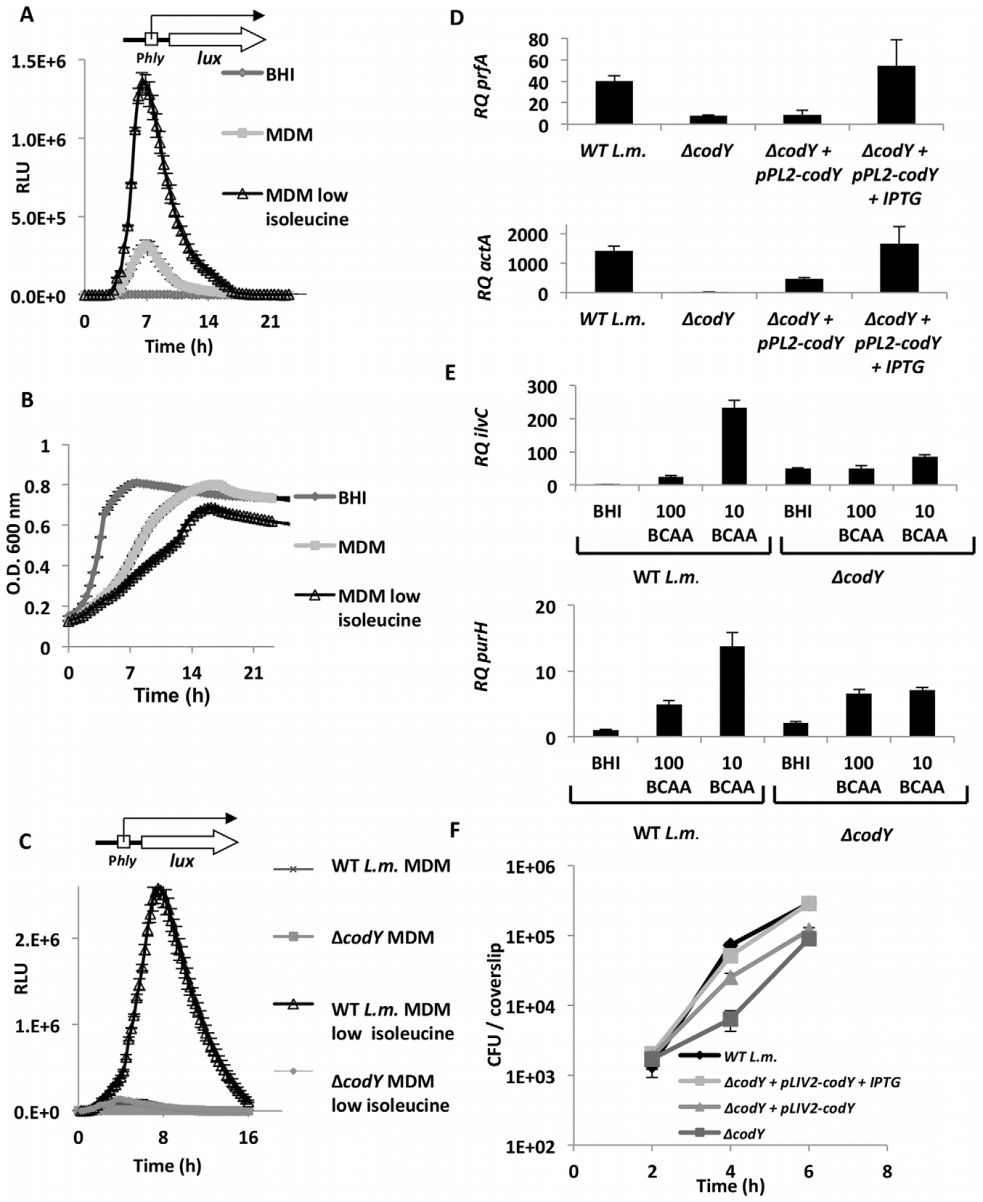
CodY regulates the transcription of virulence and metabolic genes under limiting concentrations of BCAAs. A. Relative luminescence measurements (RLU) indicating activation of *hly* promoter under growth of WT *L. monocytogenes* (harboring pPL2-P*_hly_lux* plasmid) in BHI, MDM and MDM with low concentration of isoleucine. B. Optical density measurements of the same cultures of WT *L. monocytogenes* containing pPL2-P*_hly_lux* plasmid, growing in BHI, MDM and MDM with low concentration of isoleucine. The results represent 3 independent experiments (N = 3). Error bars indicate a standard error of the mean. C. Relative luminescence measurements indicating activation of *hly* promoter in *ΔcodY* mutant and WT bacteria during growth in MDM and MDM with low concentration of isoleucine. The results represent 3 independent experiments (N = 3). Error bars indicate a standard error of the mean. D. RT-qPCR analysis of *prfA* and *actA* transcription levels during growth in MDM with low levels of isoleucine in the *ΔcodY* mutant, WT bacteria and *ΔcodY* complemented strain harboring pPL2-*codY* plasmid with and without IPTG. Levels are represented as RQ, relative to BHI and normalized to *rpoD* mRNA. The data represent 3 independent experiments (N = 3). Error bars indicate a 95% confidence interval. E. RT-qPCR analysis of *ilvC* and *purH* transcription levels in *ΔcodY* mutant and WT bacteria during growth in BHI and in MDM media with reduced concentrations of BCAAs: 100 µg/ml and 10 µg/ml. Represented as RQ, relative to BHI and normalized to *rpoD* mRNA. The data represent 3 independent experiments (N = 3). Error bars indicate a 95% confidence interval. In all experiments (A–E) overnight precultures were grown in MDM. F. Intracellular growth curve of the *ΔcodY* mutant, WT bacteria and the *ΔcodY* complemented strain (with and without IPTG) in primary BMD macrophage cells. The data represent 3 independent experiments (N = 3). Error bars indicate a standard error of the mean.

Next we tested whether the induction of the virulence genes requires low concentrations of all or some of the identified nutrients. When WT *L. monocytogenes* was grown in MDM depleted of each nutrient or combinations of nutrients, we found that limited concentrations of BCAAs is the signal for inducing expression of virulence genes (Figure 4C). Among the three BCAAs, isoleucine was found to be the most important for induction of the virulence genes, while leucine and valine presented only a minor contribution (Figure 4D). To explore the dynamic of this response we followed the induction of *hly* gene (encoding for LLO toxin) during growth in BHI, MDM and MDM with reduced concentration of isoleucine, using a *lux* reporter system. Wild type bacteria were conjugated with an integrative plasmid containing *lux* operon under the regulation of the *hly* promoter (pPL2-P*_hly_lux*) [40]. Bacteria were subjected to growth in the different media and parallel measurements of optical density (O.D._600 nm_) and luminescence were taken. As shown in Figure 5A, a robust induction of *hly* promoter was observed during logarithmic phase under limiting concentrations of isoleucine. Notably, this response was not observed during stationary phase in any of the tested conditions, suggesting that a specific regulatory mechanism involves the activation of the virulence genes under low concentrations of isoleucine (Figure 5A–5B). In Gram-positive bacteria, including *L. monocytogenes*, a nutrient responsive regulator that binds directly isoleucine is known and named CodY. CodY, when bound to isoleucine (and/or to GTP) represses genes that are required for adaptation to nutrient limitation [41]–[44]. Thus, we next examined whether CodY is involved in the regulation of *L. monocytogenes* virulence genes under low isoleucine concentrations. A *ΔcodY* complete gene deletion mutant was generated, conjugated with pPL2-P*_hly_lux* plasmid and tested under similar conditions. Interestingly, deletion of *codY* gene did not result in activation of *hly* transcription, as predicted by its role as a repressor, rather the induction of the *hly* promoter under isoleucine limiting conditions was strongly dependent on CodY (Figure 5C). In this experiment the *hly* promoter was activated only in WT bacteria grown in MDM with low concentrations of isoleucine. Similarly, *prfA*, the master virulence activator gene and *actA*, the actin-polymerizing gene, were also not induced in the *ΔcodY* mutant in comparison to WT bacteria. This phenotype was complemented by introducing a copy of *codY* gene to the *ΔcodY* mutant, using the chromosomal integrative pLIV2 plasmid (pLIV2-*codY*; under the control of an IPTG inducible promoter) (Figure 5D).

In light of these findings, we tested whether CodY has a similar role in the regulation of some relevant metabolic genes. To this end, the transcription levels of *ilvC* and *purH* genes were analyzed in the *ΔcodY* mutant and WT bacteria grown under varying concentrations of BCAAs. Notably, we observed that while *ilvC* and *purH* were highly induced when BCAAs were limiting (250 and 15-fold induction, respectively), this induction was largely dependent on CodY. However, under high BCAAs concentrations (*i.e.* in BHI), the *ΔcodY* mutant also exhibited up-regulation of *ilvC* and *purH*, but to a much lesser extent than under conditions of BCAAs depletion alone (50-and 3-fold induction, respectively) (Figure 5E). Overall, these findings indicate that while CodY and BCAAs generally repress the expression of metabolic genes, under BCAA limitation CodY is primarily involved in activation of both metabolic and virulence genes. Finally, analysis of the intracellular growth of the *ΔcodY* mutant in macrophage cells demonstrated that CodY is required for *L. monocytogenes* efficient intracellular replication, a phenotype that was complemented by in trans expression of *codY* gene (pLIV2-*codY*) (Figure 5F).

### CodY plays a positive role in *prfA* transcription specifically from *prfA* P1P2 promoters

**Figure 6:**
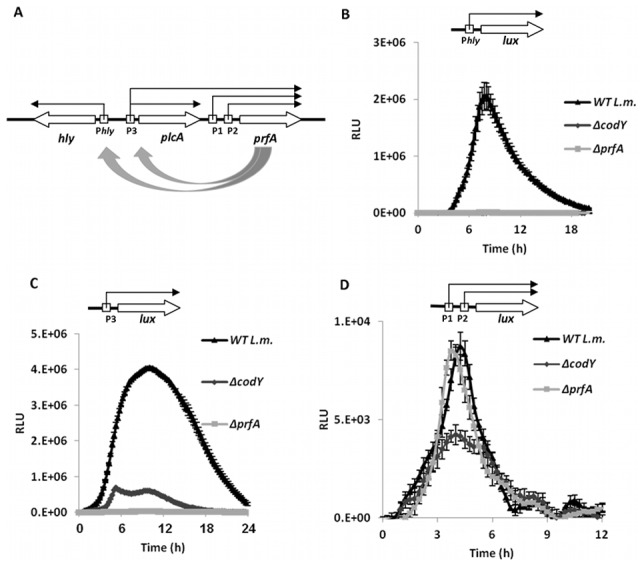
CodY is involved in a positive regulation of *prfA* transcription. A. Schematic representation of *hly*, *plcA* and *prfA* gene organization and respective promoter regions. B. Luminescence measurements of *ΔcodY*, *ΔprfA* and WT *L. monocytogenes* bacteria harboring a pPL2-P*_hly_lux* plasmid indicating P*_hly_* promotor activity during growth in MDM with low BCAA concentrations. C. Luminescence measurements of *ΔcodY*, *ΔprfA* and WT *L. monocytogenes* bacteria harboring pPL2-P3*_plcA/prfA_lux* plasmid indicating P3*_plcA/prfA_* promoter activity during growth in MDM with low BCAAs concentrations D. Luminescence measurements of *ΔcodY*, *ΔprfA* and WT *L. monocytogenes* bacteria harboring pPL2-P1P2*_prfA_lux* plasmid indicating P1P2*_prfA_* promoter activity during growth in MDM with low BCAAs concentrations. Overnight precultures were grown in MDM. The data represent 3 independent experiments (N = 3). Error bars indicate a standard error of the mean.

To study further whether CodY regulation of the virulence genes (*i.e*., *hly* and *actA* genes) is direct or mediated by PrfA, the induction of both *hly* and *prfA* promoters by CodY was assessed using the *lux* reporter system. *prfA* transcription is initiated from two distinct regions, one proximal encoding two overlapping promoters named P1 and P2, and a second distal region encoding the P3 promoter located up-stream of *plcA-prfA* genes (Figure 6A) [45]. While the latter requires PrfA itself for co-transcription of *plcA-prfA* [45], the transcription from P1P2*_prfA_* promoters is known to be PrfA-independent (Figure 6A) [46], [47]. Both *prfA* promoter regions were cloned up-stream to the *lux* operon in the pPL2-*lux* plasmid, resulting in pPL2-P1P2*_prfA_lux* and pPL2-P3*_plcA/prfA_lux*. Next, these plasmids and pPL2-P*_hly_lux* were introduced, separately, to *ΔprfA, ΔcodY* and WT bacteria and promotor activities were measured under BCAAs limiting conditions (*i.e.*, during growth in MDM media with low concentrations of BCAAs). Notably, while induction of *hly* and P3 promoters required both PrfA and CodY (Figure 6B–6C), the induction of P1P2*_prfA_* promoters was dependent solely on the *codY* gene (Figure 6D). These observations led us to conclude that CodY plays a positive role in the regulation of PrfA expression via the P1P2 promoters, while its role in the regulation of *hly* and *plcA-prfA* genes is mediated by PrfA itself. Overall the data presented here clearly demonstrate that CodY is a critical factor controlling PrfA expression linking *L. monocytogenes* metabolism and virulence.

## Discussion

In this study we examined the metabolism of the human bacterial pathogen *L. monocytogenes* during infection using both a standard bioinformatics pathway enrichment analysis and an integrative computational analysis of transcriptome data using the iMAT tool. This analysis yielded the prediction of metabolic pathways that are highly active and inactive during intracellular growth of *L. monocytogenes*, inferring the nutrients availability within the cytosolic niche. We validated the contribution of the predicted active pathways to *L. monocytogenes* growth by generating gene deletion knockouts and monitoring replication rates of these mutants in primary macrophages cells. Importantly, we used this information to search for metabolic requirements that control *L. monocytogenes* virulence. We found that low concentrations of BCAAs signal the bacteria for its intracellular location and that the isoleucine responsive regulator, CodY, is responsible for the up-regulation of *L. monocytogenes* virulence genes under these conditions.

The metabolism of *L. monocytogenes* during intracellular growth differs from its metabolism when growing in rich media. Specifically, this study revealed 12 metabolic pathways to be differentially active during infection. In accordance with previous reports the glycerol utilization pathway was identified as active during intracellular growth [10], [17]. However, it is known that *L. monocytogenes* can utilize additional carbon sources and most likely switch between them according to cytosolic availability. During residence in the host cytoplasm, *L. monocytogenes* scavenge for amino acids and vitamins. While most amino acids are acquired from the host [48], we confirmed, and in the case of histidine discovered, that several amino acid biosynthesis pathways are important for bacterial growth during infection. The requirement for arginine biosynthesis by *L. monocytogenes* likely reflects the low concentrations of this amino acid in host cytosol, as this amino acid is conditionally essential in mammalian cells [49]. In addition, arginine biosynthesis could serve to generate a nitrogen pool, since arginine can be degraded into citrulline and ammonia by arginine deiminase [21], [50]. The requirements for BCAA and histidine biosynthesis could be explained by the fact that these amino acids are not produced by human cells and therefore cytosolic concentrations could be limiting for pathogenic bacteria [49], [51]. Support for this premise comes from studies of another cytosolic pathogen, *Burkholderia pseudomalleus*, similarly found to require the BCAA and histidine biosynthesis pathways for intracellular growth [52], [53].

We show here that *L. monocytogenes* require an active histidine biosynthesis pathway for efficient intracellular growth. The HisC enzyme functions also in the synthesis of aromatic amino acids; however these amino acids were shown to be non-essential for *L. monocytogenes* intracellular replication [18]. Briefly, although the common aromatic compounds biosynthesis pathway was considered initially to be essential for *L. monocytogenes* intracellular growth, an elegant study by Goebel and colleagues delineated that the growth defect of *aro* mutants during infection is not due to the lack of aromatic amino acids, but rather to a requirement for menaquinone synthesis. Menaquinone takes part in the respiratory electron chain and this finding explained why the common aromatic compounds biosynthesis pathway is essential during aerobic growth of *L. monocytogenes* in rich medium but not under anaerobic conditions [18]. Notably, these observations point indirectly to the presence of oxygen in the host cytosol at a level sufficient for aerobic respiration. In accordance, iMAT analysis predicts the uptake of O_2_ and efflux of CO_2_ by *L. monocytogenes* during intracellular growth (Table S3).

The requirement for an active purine (adenine) biosynthesis pathway during *L. monocytogenes* intracellular growth is in accordance with a similar constraint demonstrated by other intracellular pathogens, such as *Brucella melitensis*, *Mycobacterium tuberculosis, Bacillus anthracis* and *Burkholderia pseudomallei*, and suggests limiting concentrations of purines in mammalian cells [53]–[56]. Of note, iMAT analysis identified the *de novo* purine biosynthesis pathway as induced during infection whereas conventional gene expression analysis failed to do so, implying of post-transcriptional up-regulation in this pathway. The iMAT prediction was validated as the Δ*purH* mutant displayed impaired growth in macrophages.

In addition to the discovery that histidine biosynthesis is required for *L. monocytogenes* intracellular growth, we show here for the first time that the rhamnose utilization pathway is induced and important during infection. This finding relates to an old observation that rhamnose fermentation could serve as a marker to distinguish between pathogenic and non-pathogenic isolates of *L. monocytogenes* [57]. As animals do not synthesize rhamnose, it is unlikely that rhamnose utilization provides an alternative carbon source during intracellular bacterial growth. However, rhamnose is an important and abundant carbohydrate in the cell wall of many bacterial species, including *L. monocytogenes* [58], [59], and accordingly is involved in several cell envelope processes. Indeed, an *Escherichia coli ΔrhaB* mutant displays higher resistance to cell wall antibiotics [60], supporting the hypothesis that rhamnose catabolism and turnover affect cell wall composition. Notably, a *Bacillus anthracis* mutant defective in dTDP-rhamnose synthesis was shown to adhere less to macrophages [61] and subsequently, it was reported that the macrophage CD14-MacI complex binds rhamnose residues and promotes bacterial internalization [62]. In this study we established that the *L. monocytogenes rhaB* gene encoding rhamnulose kinase is transcriptionally up regulated a 100-fold during intracellular growth. In *L. monocytogenes* rhamnose residues are known to decorate teichoic acids and were suggested to be antigenic determinants [58]. In light of our finding that the rhamnose utilization pathway is induced during infection, it is tempting to speculate that during intracellular growth *L. monocytogenes* modifies its teichoic acids to evade recognition and avoid triggering the innate immune system. The observed metabolic shift in rhamnose catabolism could directly affect the composition of the cell wall and in this way, bacterial survival during infection. Future studies are necessary to delineate why this pathway is actively required during *L. monocytogenes* intracellular growth. Overall, our findings that several amino acid biosynthesis pathways are required for growth of *L. monocytogenes* in macrophages imply that these essential metabolites might be limited in the host cytosol or that the bacteria do not transport them efficiently during intracellular growth. Differentiation between these possibilities requires further studies. It will also be important to discover if these nutrients are limiting in the absence of infecting bacteria or alternatively, are actively depleted from macrophage cytosol as a mechanism to prevent bacterial replication.

Pathogenic bacteria must express virulence factors to exploit nutrients within their host therefore it is not surprising that metabolism and virulence are closely linked. This intimate relationship is best demonstrated by the finding that intracellular carbon sources trigger up regulation of PrfA [12], [63], and thus promote escape into the cytosol and spread to neighboring cells. Here we provide an additional regulatory mechanism connecting intracellular metabolism and virulence, *i.e.*, that low availability of BCAAs, and primarily of isoleucine, leads to the induction of PrfA and in turn, of PrfA-regulated genes. The isoleucine binding transcription regulator, CodY, is directly linked to this response and, as shown, responsible for a positive regulation of *prfA*. Notably, biosynthesis of BCAAs lies at the crossroads of bacterial metabolism. The synthesis of valine and leucine requires the α-keto acid pyruvate and an amino group from glutamate, and thus is dependent on both glycolysis and the TCA cycle. Furthermore, isoleucine is synthesized from α-ketobutyrate, which is required for sulfur metabolism. Thus BCAA levels indicate the overall carbon, nitrogen, and sulfur metabolic status of the bacteria [64]. In other Gram-positive bacteria CodY was demonstrated to regulate genes involved in diverse processes such as adaptation to starvation, sporulation, biofilm and virulence [43], [65]. Although initial studies indicated a role for CodY as a general repressor (when bound to GTP and/or BCAA), later reports in *Bacillus subtilis* and *Streptococcus pneumonia* have demonstrated that it can also function as a transcriptional activator [66]–[68]. Accordingly, in some Gram-positive pathogens the expression of virulence genes was shown to be diminished in CodY null mutants [68]–[72]. In *L. monocytogenes*, CodY was shown to repress genes involved in amino acid metabolism, nitrogen assimilation and sugar uptake, and was suggested to affect virulence via a functional association with RelA [42]. Our data support the notion that CodY plays a role as repressor of metabolic genes in the presence of BCAAs. However, in this study we show that in the absence or under trace amounts of BCAAs, CodY is important for the activation of virulence genes and metabolic genes necessary for intracellular growth. We demonstrated that CodY specifically affects the expression of *prfA* via the proximal P1P2 promoters. It is still unclear whether CodY directly interacts with the *prfA* promoters or that this effect is mediated indirectly by another CodY-regulated protein. In summary, we propose that there are several regulatory connections between *L. monocytogenes* intracellular metabolism and virulence, with BCAAs and carbon source availability playing a key role in the cytosolic nutrient signature that signals the bacteria for their intracellular location.

This study is the first to address directly the ability of iMAT to serve as a tool to study the metabolic requirements of microorganisms in their natural habitat. We establish that iMAT analysis provides a more comprehensive overview of the activity of the metabolic network of an organism in a given condition. Yet, model-based analyses such as iMAT still suffer from limitations, primarily due to incomplete annotation. In addition, metabolic virulence factors like Hpt and LplA1 [13], [20] can be overlooked, as these are typically not co-regulated with a metabolic pathway. Nevertheless, the approach we presented here can be applied to other bacterial pathogens to reveal novel insights concerning pathogen specific metabolic pathways, and expand our understanding of host-pathogen metabolic interactions. This study highlights the concept that bacterial pathogens have not only acquired dedicated metabolic virulence factors but also changed the regulation of their core metabolism to be able to grow inside their hosts and trigger the virulent response in the right time; further support for the emerging view that pathogen metabolism and virulence are closely interlinked.

## Materials and Methods

### Ethics statement

The use of animals in this study was limited to preparation of bone marrow derived macrophages from mice. Experimental protocols were approved by the Tel Aviv university Animal Care and Use Committee (L-09-008) according to the Israel Welfare Law (1994) and the National Research Council guide (Guide for the Care and Use of Laboratory Animals 2010).

### Bacterial strains and growth media

*L. monocytogenes* 10403S was the wild type strain (WT) and served as the parental strain when generating gene deletion mutants (Table 2). *E. coli* XL- 1 Blue (Stratagene) was used for vector propagation and *E. coli* SM-10 strain [73] was used for plasmid conjugation to *L. monocytogenes*. The full list of the strains and plasmids used in this study are described in Table S5. Primers used in this study are described in Table S6. *L. monocytogenes* bacteria were grown at 37°C with agitation in brain heart infusion (BHI) as rich medium or in minimal defined medium (MDM), which is identical to the improved minimal media (IMM) described in [36]. MDM medium includes: phosphate buffer, 0.41 mg/ml magnesium sulfate, 10 mg/ml glucose, 100 µg/ml of each amino acid (*i.e.*, leucine, isoleucine, valine, methionine, arginine, histidine, tryptophan, glutamate, cysteine and phenylalanine), 600 µg/ml glutamine, 0.5 µg/ml biotin, 0.5 µg/ml riboflavin, 20 µg/ml ferric citrate, 1 µg/ml para-aminobenzoic acid, 5 ng/ml lipoic acid, 2.5 µg/ml adenine, 1 µg/ml thiamine, 1 µg/ml pyridoxal, 1 µg/ml calcium pantothenate and 1 µg/ml nicotinamine. For growth under limiting concentrations of nutrients, MDM was freshly made with 10-fold less of the indicated nutrients: BCAAs, histidine, arginine, adenine, phenylalanine and tryptophan (resulting in a final concentration of 10 µg/ml for amino acids and 0.25 µg/ml for adenine).

### Bacterial infection of macrophages

Bone marrow derived macrophages (BMDM) were used for infection experiments and were isolated from 6–8 week old female C57/BL6 mice (Harlan laboratories) as described previously [74]. BMDM were cultured in DMEM based media supplemented with 20% fetal bovine serum, sodium pyruvate (1 mM), L-glutamine (2 mM), b-Mercaptoethanol (0.05 mM), and M-CSF (L929-conditioned medium). Approximately 8×10^6^ *L. monocytogenes* were used to infect 2×10^6^ macrophage cells seeded in a 60 mm Petri dish, resulting in 1–2 bacteria per cell. Thirty minutes after infection, macrophage monolayers were washed three times with PBS and fresh media added. At 1 hour post infection (h.p.i.) gentamicin (50 µg/ml) was added to limit bacterial extracellular growth. Intracellular growth was evaluated as follows. Macrophages were seeded on 13 glass cover slips in a 60 mm plate. At each time point three cover slips were removed and transferred to 2 ml of double-distilled sterile water, which released intracellular bacteria. Then serial dilutions of this 2 ml were plated on BHI plates and colony-forming units (CFUs) counted the next day. Phagosomal escape assay was performed as previously described [75], WT and metabolic mutants of *L. monocytogenes* expressing GFP (pPL2-GFP integrative plasmid) [65] were used to infect BMDMs on 20 mm slides. Cells were fixed at 2.5 h.p.i. with 4% paraformaldehyde-PBS and permeabilized with Triton X-100. Actin was stained with rhodamine phalloidin (Biotium), and DNA with DAPI containing Vectashield mounting media. Images were taken using Zeiss LSM 510-META confocal microscope.

### Bacterial RNA purification and microarray analysis

RNA was harvested from bacteria growing mid-exponentially in BHI medium and from bacteria growing inside macrophages 6 h.p.i. (at which time the bacteria are cytosolic) as described previously [76]. Briefly, bacteria were harvested by filtration and the filters were frozen rapidly in liquid nitrogen. Later, filters were washed and bacterial RNA isolated using phenol-chloroform extraction. Bacterial RNA was amplified using MessageAmp™ II Bacteria Prokaryotic RNA Kit (Ambion). Microarray analysis was performed as described previously [74]. *L. monocytogenes* microarrays were printed at the Microarray Core Facility of University of California San-Francisco, using the *L. monocytogenes* oligo set designed and provided by The Institute for Genomic Research (TIGR) [76]. Statistical analysis was performed using the Statistical Analysis of Microarrays (SAM), with the false discovery rate set to 1%. Experiments were performed in duplicates. The values represent two biological repeats (N = 2).

### Quantitative real-time PCR analysis

For validation of microarray data the intracellular transcription levels of bacterial metabolic genes were analyzed using real-time quantitative PCR (RT-qPCR) at 6 h.p.i. For the analysis of metabolic genes and virulence genes during growth in BHI medium, MDM and MDM with limiting concentrations of nutrients, RNA was harvested in mid log growth phase at O.D._600_ of 0.35. In all cases precultures were grown in MDM media overnight prior to the experiments. One microgram (1 µg) of RNA was reverse transcribed to cDNA using the High Capacity reverse transcription kit (Applied Biosystems). RT-qPCR was performed on 10 ng of cDNA using SYBER Green in a Step-one Plus real time PCR system (Applied Biosystems). The transcription level of each metabolic gene was normalized to that of a reference gene: 16S rRNA in the intracellular experiments and *rpoD* mRNA in the minimal medium experiments. Statistical analysis was performed using the StepOne™ V2.1 software. RT-qPCR primers are described in Table S6.

### Analysis of metabolic pathways (gene expression and iMAT)

For the gene expression analysis, gene expression levels from the microarray analysis were discretized to 1, 0 or −1 for each gene based on a twofold change in expression in intracellular bacteria *vs.* BHI grown bacteria. The metabolic reactions and pathways of *L. monocytogenes* were obtained from the genome scale automated metabolic model [31] using PERL scripts. Given the gene expression measurement, the activity state of each enzyme/reaction was determined based on the gene-reaction mapping embedded in the metabolic model, in a standard manner. Pathway based enrichment was computed in a standard bioinformatic manner using a hypergeometric distribution, assigning an enrichment P-value for each pathway for being highly active or inactive. For iMAT analysis, the discretized gene expression levels were incorporated into the metabolic model to predict a set of high and low activity reactions (including post-transcriptional regulation predictions). The iMAT algorithm was described elsewhere [33]. Briefly, a mixed integer linear programming (MILP) problem is solved in order to find a steady-state reaction flux distribution that maximizes the number of reactions whose activity is consistent with their expression state. Subsequently, this list of active/inactive reactions was again analyzed for pathway enrichment in a standard manner, as above. The uptake reactions composing the simulated medium were selected from the pool of available exchange reactions present in the metabolic model, based on a systematic literature search in the Human Metabolome Database. The upper bounds on the set of uptake reactions selected (i.e. allowed import fluxes, measured in mmol h^−1^ g^−1^ of dry weight, Table S2) were calibrated based on *in silico* flux balance analysis simulations of bacterial replication, best representing the experimentally observed bacterial generation time. In both analyses, a correction for multiple hypotheses was done using the false discovery rate (FDR) method. Metabolic pathways with less than 3 reactions were neglected in the analysis. For statistical analysis of predictions accuracy, hypergeometric tests were applied to compare the predictions of both methods to a literature-generated database of metabolic pathways considered essential or non-essential during intracellular growth of *L. monocytogenes* (Table S4).

### Generation of *L. monocytogenes* gene deletion mutants and complementation strains

All in-frame deletions generated in this work were constructed using *L. monocytogenes* 10403S strain as the parental strain. Upstream and downstream regions of selected genes were amplified using Phusion DNA polymerase and cloned into pKSV7oriT vector [77]. Cloned plasmids were sequenced and conjugated to *L. monocytogenes* using *E. coli* SM-10 strain. *L. monocytogenes* conjugants were then grown at 41°C for two days on BHI with chloramphenicol to promote plasmid integration into the bacterial chromosome by homologous recombination. For plasmid curing, bacteria were passed several times in fresh BHI without chloramphenicol at 30°C to allow plasmid excision via the generation of an in-frame deletion. Bacteria were then seeded on BHI plates and chloramphenicol sensitive colonies picked for validation of gene deletion by PCR. Complemented strains of deletion mutants were generated by introducing *in trans* a copy of the deleted gene under the control of its native promoter using the pPL2 integrative vector. For complementation of the *codY* gene, the integrative pLIV2 plasmid was used, in which *codY* was cloned under the control of an IPTG inducible promoter [78].

### Construction and growth of *lux* reporter strains

For luminescence assays, a plasmid harboring the *lux* reporter system was used (pPL2-*lux*) [40]. For studying the regulation of the *hly* promoter pPL2-P*_hly_lux* was used and was a gift from Colin Hill [40]. For construction of pPL2-P1P2*_prfA_lux* and pPL2-P3*_plcA/prfA_lux* reporter plasmids, the intragenic regions upstream of the *prfA* and *plcA* genes were cloned into the pPL2-*lux* plasmid. *L. monocytogenes* precultures were grown in BHI or MDM media (according to experimental conditions) overnight and then diluted to O.D._600_ of 0.03 in fresh media as indicated. Bacteria were grown in a Synergy HT Biotek plate reader at 37°C. O.D._600 nm_ and luminescence measurements were taken every 15 min.

## Supporting Information

Figure S1 A. Growth of WT *Listeria monocytogenes* and metabolic mutants in MDM. B. Growth of WT *L. monocytogenes*, Δ*hisC* and Δ*hisC*+pPL2-*hisC* strains in MDM without histidine. C. Growth of WT *L. monocytogenes*, Δ*ilvC* and Δ*ilvC*+pPL2-*ilvC* strains in MDM without BCAAs. D. Growth of WT *L. monocytogenes*, Δ*argD* and Δ*argD*+pPL2-*argD* strains in MDM without arginine. E. Growth of WT *L. monocytogenes, ΔpurH* and *ΔpurH*+pPL2-*purH* strains in MDM without adenine F. Growth of WT *L. monocytogenes, ΔrhaB* and Δ*rhaB*+pPL2-*rhaB* strains in MDM with 10 mg/ml of L-rhamnose instead of D-glucose. The results represent 3 independent experiments (N = 3). Error bars represent standard error of the mean.
(TIF)

Figure S2 Growth of WT *L. monocytogenes* and the metabolic mutants in the rich lab media BHI. The results represent 3 independent experiments (N = 3). Error bars represent standard error of the mean. The *ΔaroE* mutant that exhibited a growth defect was complemented by introducing the *aroE* gene on pPL2.
(TIF)

Figure S3 Intracellular growth curves of the metabolic mutants and their complementation strains. A. Intracellular growth curves of WT *L. monocytogenes*, Δ*purH* and Δ*argD* mutants and their complemented strains. B. Intracellular growth curves of WT L. monocytogenes, Δ*aroE* and Δ*ilvC* mutants and their complemented strains. The results are representative of 3 independent experiments (N = 3). Error bars represent standard error of the mean.
(TIF)

Figure S4 Growth curves of WT *L. monocytogenes* in BHI, MDM and in MDM with reduced (Low) concentrations (10 fold less) of Arg, His, Ade and BCAAs. Precultures were grown in MDM overnight. For RNA extraction, the bacteria were harvested at O.D. of 0.3, which in all cases represented logarithmic growth. The results represnt two independent expreiments (N = 2). Error bars represent standard error of the mean.
(TIF)

Table S1 Gene expression analysis of intracellular *L.m*. vs. BHI grown *L.m*.
(XLSX)

Table S2 The modified metabolic model of *L. monocytogenes* and stimulated medium used in this study.
(XLSX)

Table S3 Metabolites confidently predicted by iMAT to be imported or exported by intracellular *L. monocytogenes*.
(XLSX)

Table S4 Literature based “gold standard” of metabolic pathways that are essential or non-essential to *L. monocytogenes* intracellular growth.
(XLSX)

Table S5 List of strains and plasmids used in this study.
(XLSX)

Table S6 List of Primers used in this study.
(XLSX)
