# Supplementary figures and images for: Integrative Genomic Analysis Identifies Isoleucine and CodY as Regulators of Listeria monocytogenes Virulence
Source: PLoS Genet. 2012 Sep 6;8(9):e1002887. doi: 10.1371/journal.pgen.1002887 (PMC3435247; doi:10.1371/journal.pgen.1002887)

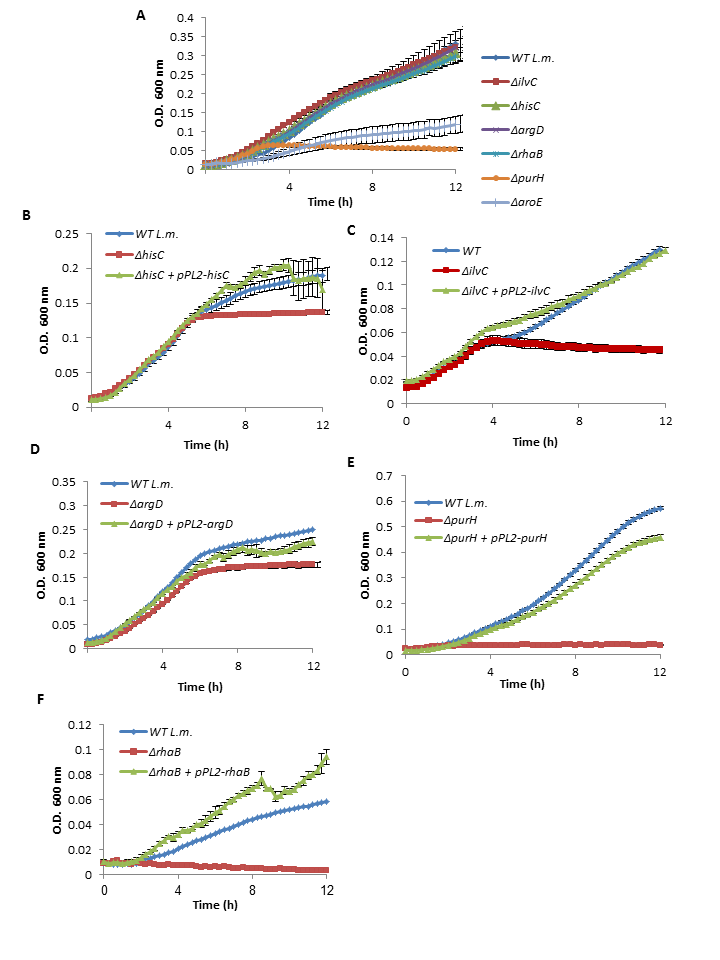

Supplement: Figure S1 — A. Growth of WT Listeria monocytogenes and metabolic mutants in MDM. B. Growth of WT L. monocytogenes, ΔhisC and ΔhisC+pPL2-hisC strains in MDM without histidine. C. Growth of WT L. monocytogenes, ΔilvC and ΔilvC+pPL2-ilvC strains in MDM without BCAAs. D. Growth of WT L. monocytogenes, ΔargD and ΔargD+pPL2-argD strains in MDM without arginine. E. Growth of WT L. monocytogenes, ΔpurH and ΔpurH+pPL2-purH strains in MDM without adenine F. Growth of WT L. monocytogenes, ΔrhaB and ΔrhaB+pPL2-rhaB strains in MDM with 10 mg/ml of L-rhamnose instead of D-glucose. The results represent 3 independent experiments (N = 3). Error bars represent standard error of the mean. (TIF) [file pgen.1002887.s001.tif]
